# Waste-Based Ceramsite for the Efficient Removal of Ciprofloxacin in Aqueous Solutions

**DOI:** 10.3390/ijerph20065042

**Published:** 2023-03-13

**Authors:** Juan Qin, Yeting Fang, Jian Shi, Chiharu Tokoro, Mauricio Córdova-Udaeta, Keishi Oyama, Juncheng Zhang

**Affiliations:** 1 Nantong Key Laboratory of Intelligent and New Energy Materials, School of Chemistry and Chemical Engineering, Nantong University, Nantong 226019, China; 2 Analysis and Testing Center, Nantong University, Nantong 226019, China; 3 Faculty of Science and Engineering, Waseda University, 3-4-1 Okubo, Shinjuku-ku, Tokyo 169-8555, Japan; 4 Faculty of Engineering, University of Tokyo, 7-3-1 Hongo, Bunkyo-ku, Tokyo 113-8656, Japan; 5 Department of Science and Engineering, Aoyama Gakuin University, Sagamihara 252-5258, Japan

**Keywords:** waste-based ceramsite, ciprofloxacin, adsorption, flocculation, regeneration

## Abstract

Ciprofloxacin (CIP), a compound with bioaccumulation toxicity and antibiotic resistance, is frequently detected in water at alarming concentrations, which is becoming an increasing concern. In this study, a low-cost ceramsite was developed from industrial solid wastes through sintering to remove CIP from wastewater. The effects of adsorbent dosage, initial pH, contact time, initial CIP concentration, and temperature were explored. More than 99% of CIP (20–60 mg/L) was removed at around pH 2–4 by the ceramsite. The kinetic data fitted well with the pseudo-second-order model, revealing that chemisorption was the main rate-determining step. The isotherm data was better described by the Freundlich model, suggesting that CIP was removed by the formation of multiple layers on the heterogeneous surface. Moreover, the removal efficiency was practically higher than 95% during five regeneration cycles, when different regeneration methods were used, including calcination, HCl, and NaOH washing, indicating that the ceramsite exhibited outstanding reusability in removing CIP. The primary mechanism of CIP removal by the ceramsite was found to be the synergism of adsorption and flocculation, both of which depended on the release of Ca^2+^ from the ceramsite. In addition, strong Ca-CIP complexes could be formed through surface complexation and metal cation bridging between Ca^2+^ and different functional groups in CIP.

## 1. Introduction

Ciprofloxacin (CIP) is a second-generation fluoroquinolone antibiotic, which was developed by Bayer A.G. in 1983 [1]. It contains a fluorine atom (F) at position six in the quinoline group, making it effective against Gram-negative bacteria and some Gram-positive bacteria [2]. Therefore, CIP is widely applied in the treatment of various infections in humans and animals, such as skin, respiratory tract, intra-abdominal, and urinary tract infections [3,4]. As reported by Proceedings of the National Academy of Sciences, global antibiotic production and consumption are increasing dramatically, and it is estimated that global antibiotic consumption will reach 69.6 billion doses by 2030, which is 200% higher than the consumption in 2015 [5]. As one of the most commonly used broad-spectrum antibiotics, CIP has been frequently detected at alarming concentrations in various water environments, including surface water (0.1–100 ng/L) [6], urban wastewater (100 ng/L–332.154 mg/L) [7], and wastewater from drug manufacturers and hospitals (3–100 mg/L) [8,9]. The existence of the F atom in the CIP structure makes it a stable contaminant in water [10]. Furthermore, CIP is a bioactive compound that can provoke and accelerate the growth of antimicrobial resistant genes (AMRs) in water, so it may pose serious risks to human and animal health [11]. O’Neill has predicted that if no responsible actions are taken to efficiently separate antibiotics and AMRs from water resources, the number of deaths due to AMRs would increase to more than 10 million per year by 2050 [12].

The removal of organic compounds from wastewater is normally a result of different processes [13,14,15]. Numerous techniques, such as biodegradation [16], photodegradation [4,10,11], advanced oxidation [17,18], adsorption [3,19], membrane separation [20], and flocculation [19,21], have been adopted to remove CIP. Among these, adsorption has attracted growing attention due to its flexibility, high efficiency, and low operational cost [22]. The effectiveness of adsorbents and adsorption conditions are key for removal efficiency and adsorption mechanism. Recently, novel adsorbents designed for CIP removal have focused mainly on chitin/chitosan-based materials, such as chitin–biocalcium [19], chitin/graphene oxide [23], MgO/chitosan [24], Al/Cu-chelated chitosan [25], Ni/NiO doped chitosan-cellulose [26], and hydrophobic chitosan flocculants [27]. In addition, nanomaterials, such as Mg(OH)_2_/MgO nanorods [28], cyclodextrin nano-sponges [29], and nano-sized polystyrene plastic [30], have also been reported in the literature to remove CIP. On the other hand, it has been demonstrated that CIP can form strong metal–CIP complexes with double cations, such as Ca^2+^ and Mg^2+^ in animals, seawater, and soil minerals [13,19,24]. The formation of these complexes can significantly decrease the gastrointestinal adsorption of CIP and cause a loss in detectability and antibacterial activity in the environment [13]. Moreover, minerals, such as Fe/Al/Si oxides with both positive and negative surface charge, can provide the major sorption sites for CIP, even when present in limited quantity [31]. Inspired by these findings, a novel waste-based ceramsite, prepared in our previous work [32], which can release Ca^2+^ spontaneously into aqueous solutions and contains other oxides, such as SiO_2_, Al_2_O_3_, and MgO, may provide a better solution for CIP removal.

Therefore, the prime objective of this study was to assess the feasibility of removing CIP from aqueous solutions using a low-cost and environmentally friendly ceramsite prepared from industrial solid wastes, i.e., lime mud and fly ash. To achieve this, several crucial parameters, including adsorbent dosage, initial pH, contact time, initial CIP concentration, and temperature, were investigated by batch experiments. Furthermore, the effectiveness of the ceramsite in removing CIP from simulated CIP-contaminated sewage was also tested. CIP desorption was carried out using different methods, and the reusability of the ceramsite was explored. The removal process was also fitted to kinetic and isotherm models, and different characterizations were carried out to describe the mechanisms involved in CIP removal.

## 2. Materials and Methods

### 2.1. Materials and Reagents

Lime mud from Xinda Paper Industry Co., Ltd. (Xuzhou, China) and fly ash from Huaneng Nanjing Power Station (Nanjing, China) were used as raw materials to synthesize the waste-based ceramsite using a mass ratio of 1:1 and a temperature of 1050 °C. The detailed chemical composition of the raw materials and the synthetic procedure of the ceramsite were introduced in our previous study [32].

Ciprofloxacin (CIP, 98%) was purchased from Shanghai Macklin Biochemical Co., Ltd. (Shanghai, China) Hydrochloric acid (HCl, 36–38%) and sodium hydroxide (NaOH, ≥96%) were purchased from Shanghai Lingfeng Chemical Reagent Co., Ltd. (Shanghai, China) and Xilong Scientific Co., Ltd. (Shantou, China), respectively. Quartz ceramsite was purchased from Nanjing Dongji new building materials Co., Ltd. (Nanjing, China).

### 2.2. Characterization

The crystalline phases were evaluated by X-ray diffraction (XRD, D8 advance, Bruker, Karlsruhe, Germany) with CuK_α_ radiation (λ = 1.542 Å) at 40 kV. The surface chemical structures were identified by X-ray photoelectron spectroscopy (XPS, K-Alpha+, Thermo Scientific, Waltham, MA, USA). The characteristic function groups were determined by Fourier transform infrared spectroscopy (FTIR, Tensor 27, Bruker, Heidelberg, Germany). The surface microstructures were visualized by a scanning electron microscopy (SEM, Gemini SEM 300, Zeiss, Jena, Germany). The zeta potential was obtained by Zetasizer nano ZS 90 (Malvern, UK). 

The pore size distribution was identified by a mercury porosimeter (Model GT-60, Quantachrome, Boynton Beach, FL, USA). The bulk density and 24 h water absorption were detected by the Archimedes method (ASTM C373). The apparent porosity was measured according to ISO 18754. The cylinder compressive strength was tested referring to BS EN 13055 on an electronic universal tester (Model CMT5105, SANS, Shenzhen, China). In addition, a leaching test of the ceramsite was carried out according to BS EN 12457-2, and the concentration of heavy metals was measured by inductively coupled plasma mass spectroscopy (ICP-MS, Model ICP-MS 2000, Skyray, Suzhou, China).

### 2.3. Batch Experiments

Batch experiments were performed to explore the removal behavior of the waste-based ceramsite on CIP under different conditions, including adsorbent dosage, initial solution pH, contact time, initial CIP concentration, and temperature. In each experiment, 200 mL of CIP solution was mixed with the ceramsite in a 250 mL conical flask and shaken at 25 °C and 120 r/min in the absence of light. During the dosage effect experiment, the ceramsite amount was 2, 4, 6, 8, 10 g/200 mL, the initial solution pH was set to 2.0, and the CIP concentration was 60 mg/L. The dosage amount was finally fixed at 6 g/200 mL in other adsorption experiments. To investigate the effect of solution pH, the initial pH of the CIP solution (60 mg/L) was regulated to desired pH values of 1.0, 2.0, 4.0, 6.0, 8.0, and 10.0, using 1 M NaOH and HCl solutions. The initial solution pH was kept at 2.0 in other adsorption experiments. For the kinetics experiment, the CIP concentration was 60 mg/L, and the sampling time was predetermined at 0.25, 0.5, 1, 2, 3, 4, 6, 8, 10, 12, 14, and 24 h. The isotherm experiment was performed at different initial CIP concentrations (20, 40, 60, 80, 100, 200, 300, and 400 mg/L), and the sampling time was 12 h. The thermodynamic experiment was conducted at four different temperatures (15, 25, 35, and 45 °C) and two initial CIP concentrations (60 and 100 mg/L).

The feasibility of removing CIP from actual wastewater using the waste-based ceramsite was also examined. For this purpose, 2 L of the influent of a local wastewater treatment plant (WWTP) in Qidong (Nantong, China) were collected. The concentrations of some typical anions and antibiotics in the influent are shown in Table S1. A total of 60 mg/L of simulated CIP-contaminated sewage was artificially prepared by adding a certain amount of concentrated CIP solution to the collected influent.

Aqueous samples were filtered by a 0.45 μm syringe filter and then kept in a sample tube. The concentration of CIP was then measured by a liquid chromatograph (1260 Infinity, Agilent, Santa Clara, CA, USA). Other typical antibiotics were detected by a liquid chromatograph-mass spectrometer (5500QTRAP, SCIEX, Boston, MA, USA). Anions (Cl^−^, NO_3_^−^, SO_4_^2−^) were detected by an ion chromatograph (DIONEX ICS-5000+ DC, Thermo Fisher Scientific, Waltham, MA, USA). The removal efficiency (R, %) and adsorption capacity (Q_t_, mg/g) of CIP were calculated by Equations (1) and (2):

R = (C_0_ − C_t_)/C_0_ × 100% (1)

Q_t_ = (C_0_ − C_t_) × V/m (2)

where C_0_ (mg/L) is the initial CIP concentration, C_t_ (mg/L) is the CIP concentration at time t, V (L) is the solution volume, and m (g) is the mass of the ceramsite used.

To investigate the CIP removal mechanism in detail, the kinetic data of CIP removal by the ceramsite at 25 °C was fitted using the pseudo-first-order and pseudo-second-order kinetic models [22]. The mathematical equations of the two models are presented in Equations (3) and (4):

ln(Q_e_ − Q_t_) = lnQ_e_ − k_1_t (3)

t/Q_t_ = 1/(k_2_Q_e_^2^) + t/Q_e_ (4)

where t (h) is the contact time, Q_e_ (mg/g) is the adsorption capacity at equilibrium, k_1_ (1/h) is the rate constant of the pseudo-first-order model, and k_2_ [g/(mg·h)] is the rate constant of the pseudo-second-order model.

The Langmuir and Freundlich isotherm models, whose mathematical equations are presented in Equations (5) and (6), were used to fit the equilibrium data [22].

C_e_/Q_e_ = 1/(Q_m_K_L_) + C_e_/Q_m_ (5)

lnQ_e_ = lnK_F_ + nlnC_e_ (6)

where C_e_ (mg/L) is the concentration at equilibrium, Q_e_ (mg/g) and Q_m_ (mg/g) are the equilibrium and maximum adsorption capacity, K_L_ (L/mg) is the constant of the Langmuir model, K_F_ [(mg/g)/(mg/L)^1/n^] is the constant related to adsorption capacity of the Freundlich model, and n is the empirical constant related to adsorption strength of the Freundlich model.

### 2.4. Desorption and Reusability of the Waste-Based Ceramsite

In order to explore the desorption and reuse abilities of the waste-based ceramsite, an adsorption-desorption experiment was carried out for five cycles. Both 0.05 M HCl and 0.1 M NaOH were used as elution solutions to desorb the adsorbed CIP. Moreover, the adsorbed ceramsite was regenerated by calcination at a temperature of 800 °C for 50 min in a muffle furnace (KSL-1200X, Kejing, Hefei, China). In all the batch experiments described here, the initial CIP concentration was 60 mg/L, the ceramsite dosage was 6 g/200 mL, and the contact time was 12 h.

## 3. Results and Discussion

### 3.1. Characterization of the Waste-Based Ceramsite

The photograph, XRD pattern, SEM image, and pore size distribution of the ceramsite are shown in Figure S1, respectively. The XRD pattern in Figure S1b identified anorthite (CaAl_2_Si_2_O_8_), wollastonite (CaSiO_3_), and gehlenite (Ca_2_Al_2_SiO_7_) as the primary crystalline phases, indicating that the prepared ceramsite was a kind of Ca-rich material. The SEM image (Figure S1c) and pore size distribution (Figure S1d) confirmed the existence of interconnected macropores (approximately 2 μm in diameter) within the ceramsite, which could provide abundant inner space and binding sites for adsorption. The low bulk density (0.88 g/cm^3^), high water absorption (30.15%), and apparent porosity (48.57%) presented in Table S2 also indicated that the ceramsite had a porous structure. Since the ceramsite was synthesized from industrial solid wastes, a toxicity leaching test was conducted and demonstrated that the concentrations of toxic elements were within environmental standards, thus indicating that the ceramsite was environmentally friendly (Table S3). 

### 3.2. Batch experiments

#### 3.2.1. Effect of the Waste-Based Ceramsite Dosage

**Figure 1:**
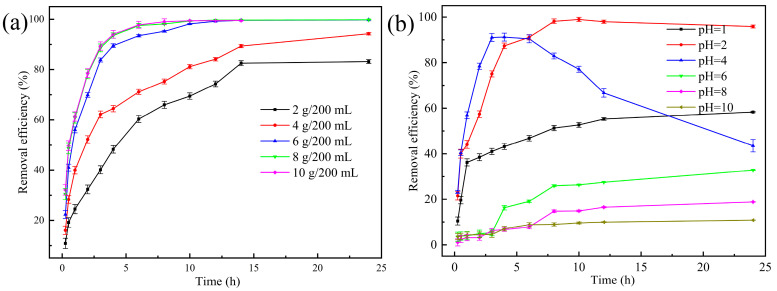
Removal efficiency of CIP at different (**a**) dosage (initial pH: 2.0; CIP concentration: 60 mg/L; temperature: 25 °C) and (**b**) initial pH (ceramsite dosage: 6 g/200 mL; CIP concentration: 60 mg/L; temperature: 25 °C).

The effect of ceramsite dosage on CIP removal (60 mg/L) was explored. As illustrated in Figure 1a, increasing the dosage from 2 g to 6 g/200 mL led to an obvious increase in the removal efficiency of CIP from 82.50% to 99.59%, accompanied by a faster removal rate. The increased dosage could contribute more active sites, so the removal efficiency was correspondingly promoted [33]. Nevertheless, no evident improvement was obtained after increasing the dosage from 6 g to 10 g/200 mL. Hence, considering both economy and high efficiency, 6 g/200 mL was determined as the optimum ceramsite dosage and applied in subsequent experiments.

#### 3.2.2. Effect of Initial pH

**Figure 2:**
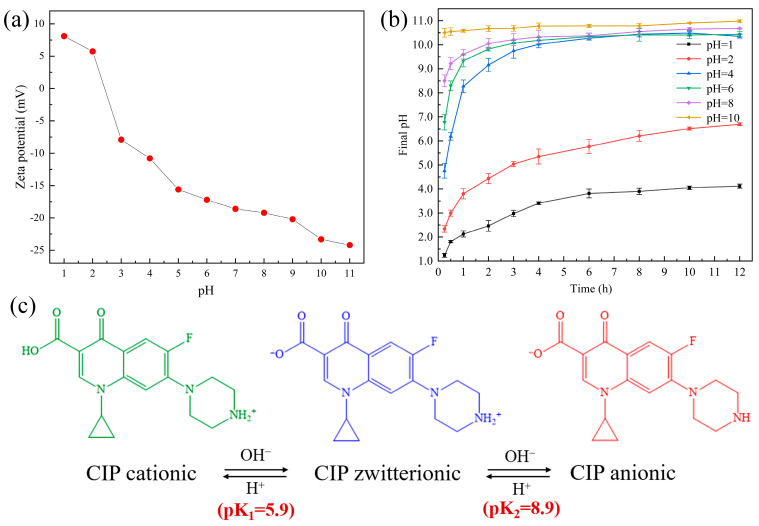
(**a**) Zeta potential of the waste-based ceramsite at different initial pH; (**b**) pH change of aqueous solutions adding the waste-based ceramsite at different initial pH (ceramsite dosage: 6 g/200 mL; temperature: 25 °C); (**c**) ionic forms of CIP.

The surface charge of adsorbents and the dominant CIP species in aqueous solutions are obviously influenced by the solution pH [28]. The zeta potential of the ceramsite is shown in Figure 2a. The surface charge became negative when the solution pH was >2. Figure 2b indicates that the ceramsite has the ability to self-adjust pH by spontaneously releasing OH^−^ into aqueous solutions to improve the pH. This ability has also been verified in our previous study [34]. Furthermore, as described in Figure 2c, the predominant form of CIP at acidic (pH < pK_1_ = 5.9), neutral (pK_1_ = 5.9 < pH < pK_2_ = 8.9), and alkaline pH (pH> pK_2_ = 8.9) is CIP^+^ (cationic), CIP^±^ (zwitterionic), and CIP^−^ (anionic), respectively [19]. Hence, the effect of initial pH on CIP removal (60 mg/L) was investigated in Figure 1b and found to be important. After 12 h of contact, the removal efficiency of CIP increased from 55.26% to 97.99% when the initial pH increased from 1 to 2, but decreased continuously to 10.83% when the initial pH increased from 2 to 10. It could be inferred that acidic pH, especially around 2, was the optimal pH condition for the ceramsite to remove CIP. The observed pH-dependent trend indicated that the interaction between CIP and the ceramsite was complex and controlled by multiple mechanisms [35]. In combination with the final pH of solutions adjusted by the ceramsite in Figure 2b, CIP was likely present in the form of CIP^+^ when the initial pH was <2, mainly in the form of CIP^±^ when the initial pH was 2–4 (being the initial contact at pH = 4), and in the form of CIP^−^ when the initial pH was >4. Considering the zeta potential of the ceramsite (Figure 2a), electrostatic attraction between CIP and the ceramsite was probably promoted as they were both zwitterionic when the initial pH was 2–4 (being the initial contact at pH = 4). However, electrostatic repulsion was not beneficial to CIP removal, as the surfaces of CIP and the ceramsite were both positively or negatively charged when the initial pH was <2 or >4. Except for electrostatic attraction, a part of the total CIP could still be removed by the ceramsite, especially when the initial pH was 1, indicating that there was another mechanism involved in the removal, which will be discussed in Section 3.3. 

#### 3.2.3. Effect of Contact Time and Adsorption Kinetics

**Figure 3:**
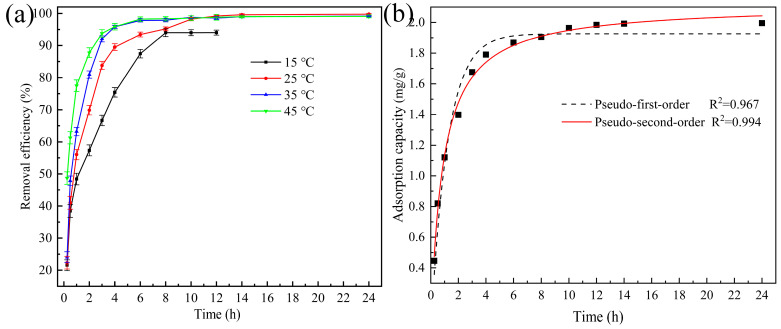
(**a**) Removal efficiency of CIP at different contact time and temperature (initial pH: 2.0; ceramsite dosage: 6 g/200 mL; CIP concentration: 60 mg/L); (**b**) kinetics analysis for CIP removal by the waste-based ceramsite.

The effect of contact time on CIP removal by the ceramsite was investigated at an initial concentration of 60 mg/L and different temperatures. As illustrated in Figure 3a, the removal efficiency of CIP rapidly increased in the first 4 h, which was attributed to the large amount of available active sites on the surface of the ceramsite. After that, the removal efficiency reached equilibrium and remained stable due to the saturation of the abovementioned active sites. Additionally, higher temperatures could promote CIP removal in the range of 15–35 °C, whereas the positive influence was not evident when the temperature was >35 °C. The non-linear plots for the pseudo-first-order and pseudo-second-order kinetic models are presented in Figure 3b, and the obtained kinetic parameters are summarized in Table S4. The results indicated that the CIP removal process using the ceramsite fitted the pseudo-second-order model better, with a higher correlation coefficient (R^2^ = 0.994). This suggested that chemical adsorption was the main rate-determining step [19,28]. According to Table S4, the Q_e_ value for CIP calculated from the pseudo-second-order model was 2.12 mg/g, which was very close to the experimental data of 2.00 mg/g. 

**Figure 4:**
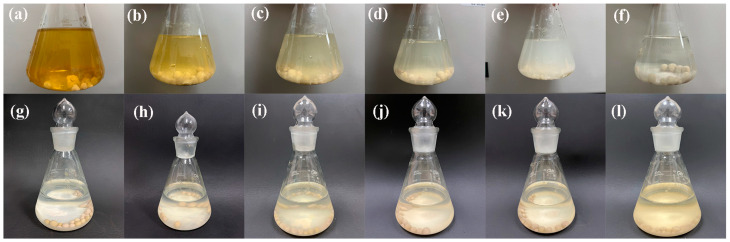
Photographs of samples at different contact time: (**a**) 0.5 h, (**b**) 2 h, (**c**) 6 h, (**d**) 8 h, (**e**) 24 h, (**f**) standing for 2 h (initial pH: 2.0; ceramsite dosage: 6 g/200 mL; CIP concentration: 60 mg/L; temperature: 25 °C); photographs of samples at different concentrations after standing for 2 h: (**g**) 20 mg/L, (**h**) 60 mg/L, (**i**) 100 mg/L, (**j**) 200 mg/L, (**k**) 300 mg/L, (**l**) 400 mg/L (initial pH:2.0; ceramsite dosage: 6 g/200 mL; temperature: 25 °C; contact time: 12 h).

Figure 4a–f exhibit photographs of the CIP removal process at different contact times. It has been reported that the color of CIP solutions deepens when the pH is <2.8 or >5.5 [36]. The initial pH of the CIP solution (60 mg/L) was 2.0, so the color was yellow in the beginning. As time passed, the pH was elevated and CIP was adsorbed by the ceramsite, correspondingly resulting in the yellow fading. During the removal process, flocs were observed and settled after standing for 2 h. Since the ceramsite could spontaneously release Ca^2+^ into aqueous solutions [34], Ca^2+^ and CIP likely formed Ca-CIP complexes through flocculation [37]. These flocs with good sedimentation properties could be easily removed from water and would not become another form of contaminant afterwards. In an actual application, other precipitates/co-precipitates, such as calcium carbonate (CaCO_3_) or a mixture of CIP-complex carbonate, might also form if carbon dioxide (CO_2_) is present in wastewater due to biologically activated degradation processes.

#### 3.2.4. Effect of Initial CIP Concentration and Isotherm Analysis Kinetics

**Figure 5:**
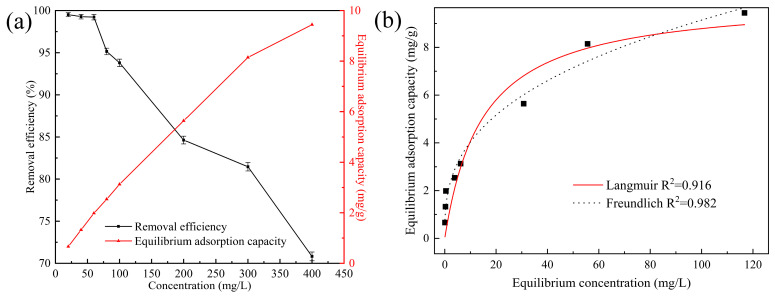
(**a**) Removal efficiency and adsorption capacity of CIP at different concentrations (initial pH: 2.0; ceramsite dosage: 6 g/200 mL; temperature: 25 °C; contact time: 12 h); (**b**) Langmuir and Freundlich isotherm models for CIP removal by the waste-based ceramsite.

The initial concentration of contaminants is an important driving force for mass transfer between solid and liquid phases during adsorption [38]. To explore the effect of initial CIP concentration on removal by the ceramsite, solutions with different initial CIP concentrations were investigated, and the removal efficiency and adsorption capacity are exhibited in Figure 5a. Increasing CIP concentration from 20 to 400 mg/L gradually decreased the removal efficiency from 99.52% to 70.82% but increased the adsorption capacity from 0.66 mg/g to 9.44 mg/g. Generally, when the concentration of contaminants is excessive, the active sites on the surface of adsorbents are promptly occupied, and the available active sites dramatically decrease, resulting in only a slight increase in the adsorption capacity [28]. However, the adsorption capacity for CIP by the ceramsite kept increasing almost linearly, indicating that there might be other removal mechanisms besides adsorption. Figure 4g–l provided photographs of the CIP removal process at different initial CIP concentrations, revealing that more and more flocs were formed and eventually settled with the increase of CIP concentration. Thus, the synergism of adsorption and flocculation might be the primary mechanism of CIP removal.

The non-linear plots for the Langmuir and Freundlich isotherm models are presented in Figure 5b, and the obtained isotherm parameters are summarized in Table S5. The experimental data were better described by the Freundlich model, with a higher correlation coefficient (R^2^ = 0.982), suggesting that CIP was removed by the formation of multiple layers on the heterogeneous surface [39]. The n parameter for the Freundlich model is generally identified as irreversible (n = 0), favorable (n *<* 1), linear (n = 1), and unfavorable (n > 1) [40]. The n value calculated in this study was 0.355, indicating that CIP adsorption by the ceramsite was a favorable process.

#### 3.2.5. Effect of Temperature

The effect of temperature on CIP removal by the ceramsite was investigated by elevating the temperature from 15 to 45 °C. At an initial CIP concentration of 60 mg/L, it was found that the higher temperature could slightly promote CIP removal, as displayed in Figure 3a. However, the majority of the previous studies have shown that lower temperatures are beneficial for CIP removal because CIP adsorption is usually spontaneous and exothermic [3,19,28]. Thus, a higher initial CIP concentration of 100 mg/L was selected for further validation, as shown in Figure S2, which also illustrated that temperature had a very minor effect on CIP removal. In addition to adsorption, it was possible that flocculation contributed to CIP removal, which was closely related to the release of Ca^2+^ from the ceramsite. Our previous study demonstrated that the release of Ca^2+^ can be accelerated by the increased temperature [34]. Considering the opposite effects of temperature on the possible processes during CIP removal, it was likely that the CIP removal efficiency was not regularly and dramatically affected by temperature conditions.

#### 3.2.6. Treatment of Actual Wastewater

**Figure 6:**
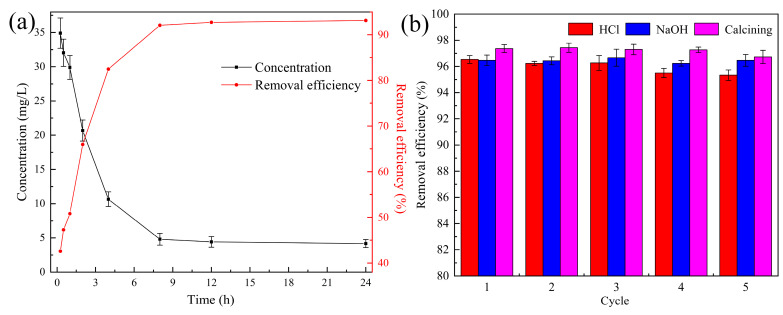
(**a**) Concentration and removal efficiency of CIP in actual wastewater after removal (initial pH: 2.0; ceramsite dosage: 6 g/200 mL; CIP concentration: 60 mg/L; temperature: 25 °C); (**b**) removal efficiency of CIP for 5 consecutive cycles using the waste-based ceramsite regenerated by different methods (initial pH: 2.0; ceramsite dosage: 6 g/200 mL; CIP concentration: 60 mg/L; temperature: 25 °C; contact time: 12 h).

To further evaluate the CIP removal performance of the ceramsite, CIP-contaminated sewage with an initial CIP concentration of 60 mg/L was simulated using the influent of a local WWTP. As shown in Table S1, the main anions of the raw sewage were Cl^−^ (541.27 mg/L), SO_4_^2−^ (37.91 mg/L), and NO_3_^−^ (0.59 mg/L), along with some typical antibiotics which were found with concentrations in the ng/L range. After 8 h of treatment, the CIP concentration decreased to 4.81 mg/L, with a removal efficiency of 92.08%, as shown in Figure 6a. Figure 3a indicated that the removal efficiency of CIP in deionized water was 95.21% under the same conditions. This similar behavior proved that the matrix found in the raw sewage hardly interfered with CIP removal by the ceramsite. In addition, these results also confirmed the extraordinary performance of the ceramsite in the treatment of real CIP-contaminated waters.

#### 3.2.7. Regeneration and Reusability

Reusability is an indispensable indicator for the evaluation of the service life of an adsorbent [41]. In this study, two kinds of eluents, i.e., 0.05 M HCl and 0.1 M NaOH, and calcination at 800 °C were, respectively, used to regenerate the ceramsite after adsorbing CIP. As depicted in Figure 6b, the ceramsite exhibited outstanding performance in removing CIP, with a removal efficiency higher than 95% during the 5 regeneration cycles. Calcination was considered the optimal regeneration method for the ceramsite due to the highest removal efficiency obtained (96.2–97.7%), and compared with HCl, the regeneration performance of NaOH was more stable in the later cycles, making it more suitable as eluent for the ceramsite. It also could be predicted from Figure 6b that the ceramsite had the ability to endure more than five regeneration cycles. In conclusion, the reusability of the ceramsite without a significant loss in the removal efficiency of CIP has great potential in terms of significantly reducing treatment costs for practical applications.

### 3.3. Mechanisms of CIP Removal by the Waste-Based Ceramsite

**Figure 7:**
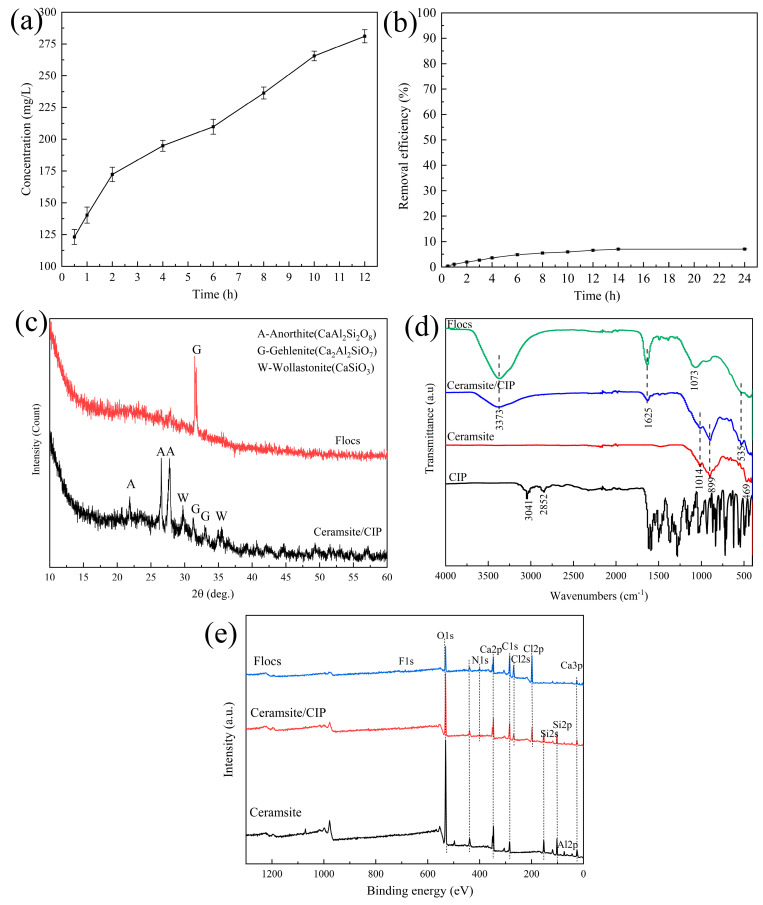
(**a**) Ca^2+^ concentration of the waste-based ceramsite released (initial pH of 2.0; ceramsite dosage: 6 g/200 mL; temperature: 25 °C); (**b**) removal efficiency of CIP by quartz ceramsite (initial pH: 2.0; ceramsite dosage: 6 g/200 mL; CIP concentration: 60 mg/L; temperature: 25 °C); (**c**) XRD patterns; (**d**) FTIR spectra; (**e**) XPS curves of the waste-based ceramsite and flocs during CIP removal.

The results of the batch experiments, kinetics, and isotherm analyses indicated that the synergism of adsorption and flocculation might control CIP removal by the waste-based ceramsite. To thoroughly investigate the removal mechanisms, the chemical composition characteristics of the ceramsite and flocs formed during CIP removal were analyzed, and the results are shown in Figure 7. Considering that the ceramsite obtained the highest removal efficiency of CIP at an initial pH of 2.0, the Ca^2+^ concentration released from the ceramsite at the same initial pH in deionized water was measured. As exhibited in Figure 7a, the Ca^2+^ concentration increased persistently to 281.08 mg/L with increasing contact time. Anorthite (CaAl_2_Si_2_O_8_), wollastonite (CaSiO_3_), and gehlenite (Ca_2_Al_2_SiO_7_) were identified as the main crystalline phases of the ceramsite in Figure S1b. Their acid resistance is as follows: CaSiO_3_ < Ca_2_Al_2_SiO_7_ < CaAl_2_Si_2_O_8_. Thus, wollastonite and gehlenite are more prone to dissolve than anorthite in an acidic environment, as shown in Equations (7) and (8), causing the release of Ca^2+^ from the ceramsite [34]. Several studies have reported that metal–CIP surface complexes can be generated in solutions [19,31,42]. Especially, a cation-bridge structure between Ca^2+^ and CIP^±^ is found to be easily formed, mainly due to the keto O group and the deprotonation of the carboxylic group in CIP molecular, with a complexation constant of 14.7 [42]. In order to evaluate the contribution of the released Ca^2+^ for CIP removal, quartz ceramsite, a common Ca-free adsorbent, was used as a control material. As shown in Figure 7b, only 7.02% of CIP was removed by quartz ceramsite under the same experiment conditions. Therefore, it was evident that CIP removal depended on the release of Ca^2+^ from the waste-based ceramsite. In addition, Ca^2+^ was continually consumed during CIP removal, and the adsorbed CIP on the surface of the ceramsite hindered the release of Ca^2+^ in the later stages, so the residual Ca^2+^ concentration during CIP removal was measured and found to be in the range of 35.33–52.61 mg/L. This concentration was not expected to severely affect water hardness.

CaSiO_3_ + H^+^ → Ca^2+^ + SiO_2_ + H_2_O (7)

Ca_2_Al_2_SiO_7_ + H^+^ → Ca^2+^ + Al^3+^ + SiO_2_ + H_2_O (8)

Figure 7c shows that there were obvious changes in the XRD patterns of the ceramsite and flocs after CIP removal. In terms of the ceramsite/CIP, most of the characteristic diffraction peaks of wollastonite and gehlenite disappeared, indicating that the structures of these two crystals were partially destroyed during CIP removal. In terms of the flocs, only a main diffraction peak corresponding to gehlenite remained, and a slight diffuse peak appeared in the range of 2θ = 15–30°, suggesting that a certain amount of an amorphous phase was generated in the flocs. This phenomenon indicated that anorthite was responsible for the chemical stability of the ceramsite, while both wollastonite and gehlenite participated in CIP removal through surface complexation or cation bridging. CIP might act as a ligand to promote the dissolution of these two crystals [19,35].

To compare the functional groups on the surface of the ceramsite before and after CIP removal, FTIR analysis was performed and is exhibited in Figure 7d. For CIP, the main characteristic peaks were observed between 1200 and 1800 cm^−1^. Specifically, the peaks at 1613 cm^−1^, 1586 cm^−1^, 1372 cm^−1^, and 1260 cm^−1^ corresponded to the stretching vibration of ketone O, the asymmetric stretching vibration of -COO^−^, the symmetric stretching vibration of -COO^−^, and the deformation vibration of O-H, respectively [28,35]. In addition, the small peak at 3041 cm^−1^ was related to the stretching vibration of O-H and N-H. For the ceramsite, the two primary peaks at 1014 cm^−1^ and 899 cm^−1^ were ascribed to the bending vibration of Si-O-Si and the symmetric stretching vibration of Si-O, respectively. After CIP removal, a broad peak at around 3373 cm^−1^ appeared in both the ceramsite and flocs, which might be caused by the bonding between O-H in CIP and Ca^2+^ released from the ceramsite. Moreover, the peak at 1613 cm^−1^ shifted to 1625 cm^−1^, and the peaks at 1586 cm^−1^, 1372 cm^−1^, and 1260 cm^−1^ almost vanished, indicating that ketone O, -COO^−^, and O-H were all involved in the removal process, possibly due to the cation bridging or surface complexation between Ca^2+^ and CIP ions [19]. For the flocs, the peak at 1014 cm^−1^ shifted to 1073 cm^−1^, and the peak at 899 cm^−1^ was barely visible when compared with the ceramsite, demonstrating that a part of Ca^2+^ was released from the ceramsite towards the solution, in order to combine with CIP and then form the flocs. Therefore, the successful adsorption and flocculation of CIP could be inferred from the abovementioned results.

In addition, XPS analysis was applied to investigate the interaction between the ceramsite and CIP, as displayed in Figure 7e. After CIP removal, two small peaks at 686 eV and 400 eV corresponding to the atomic F1s and N1s in the CIP molecular were observed in the flocs. Moreover, the response of Ca2p and Ca3p at 351 eV and 25 eV was detected in both the ceramsite and the flocs, while the response of Si2s and Si2p remained only in the ceramsite but not in the flocs. This observation revealed that Ca^2+^ released from the ceramsite was conducive to CIP removal, especially in terms of the flocculation of CIP, which was consistent with the FTIR analysis.

**Figure 8:**
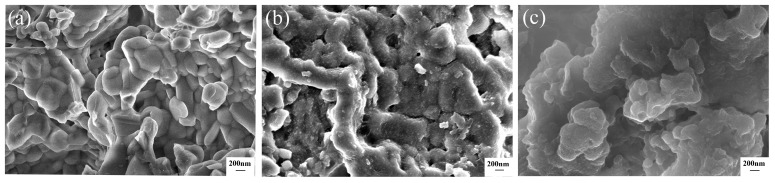
(**a**) SEM image of the waste-based ceramsite before CIP removal; (**b**) SEM image of the waste-based ceramsite after CIP removal; (**c**) SEM image of flocs after CIP removal.

The microstructures of the ceramsite and flocs during CIP removal are shown in Figure 8. Compared with the ceramsite before CIP removal in Figure 8a, it was obvious that a large number of small particles were attached to the surface of the ceramsite after CIP removal in Figure 8b. This significantly increased the surface roughness. The morphology of the flocs shown in Figure 8c was irregularly agglomerated. These findings confirmed intuitively that CIP was successfully adsorbed on the surface of the ceramsite to a certain extent, and the flocculation also contributed to CIP removal.

**Figure 9:**
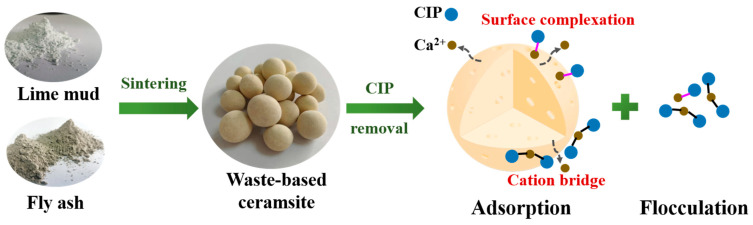
Synthesis of the waste-based ceramsite and schematic diagram of mechanisms of CIP removal.

Based on the analysis shown above, it could be concluded that the synergism of adsorption and flocculation should be the primary mechanism of CIP removal by the waste-based ceramsite, as displayed in Figure 9. Adsorption could occur in solutions of different CIP concentrations, while flocculation gradually appeared with increasing initial CIP concentration. Importantly, the adsorption and flocculation mechanisms in this study were found to be similar, mainly relying on the release of Ca^2+^ from the ceramsite. Strong Ca-CIP complexes were formed through surface complexes and metal cation bridging between Ca^2+^ and different functional groups in CIP, including ketone O, -COO^−^, and O-H.

## 4. Conclusions

In this study, a low-cost and environmentally friendly ceramsite prepared from industrial solid wastes was successfully utilized to remove CIP from aqueous solutions. Batch experiments were conducted by varying parameters, such as adsorbent dosage, initial pH, contact time, initial CIP concentration, and temperature. The results revealed that the ceramsite exhibited high removal efficiency of 70.82–99.52% for initial CIP concentrations of 20–400 mg/L and an initial pH range of 2–4. The experimental data fitted well with the Freundlich isotherm model and the pseudo-second-order kinetic model, indicating that CIP removal occurred across multiple layers on the heterogeneous surface and was dominated by chemisorption. The experiment using simulated CIP-contaminated sewage showed that the matrix of the raw sewage did not interfere with CIP removal by the ceramsite. Desorption experiments using two eluents (HCl and NaOH) and calcining at 800 °C showed that the ceramsite could be successfully regenerated up to 5 cycles with a removal efficiency of >95%, indicating its potential for practical applications in reducing treatment costs. Characterizations, including XRD, FTIR, XPS, and SEM, indicated that the synergistic mechanisms of adsorption and flocculation were responsible for CIP removal by the ceramsite, which significantly depended on the release of Ca^2+^ from the ceramsite. Strong Ca-CIP complexes could be formed through surface complexation and metal cation bridging between Ca^2+^ and different functional groups (i.e., ketone O, -COO^−^, and O-H) in CIP. Therefore, the low-cost waste-based ceramsite demonstrated great potential as an efficient adsorbent for CIP removal.

## Data Availability

Not applicable.

## Supplementary Materials

The following supporting information can be downloaded at: https://www.mdpi.com/article/10.3390/ijerph20065042/s1, Figure S1: (a) Photograph, (b) XRD pattern, (c) SEM image, and (d) pore size distribution of the waste-based ceramsite; Figure S2: Removal efficiency of CIP (100 mg/L) at different temperature; Table S1: Concentrations of typical anions and antibiotics in the influent of a local sewage treatment plant; Table S2: Physical properties of the waste-based ceramsite; Table S3: Results of leaching test for the waste-based ceramsite; Table S4: Kinetic model parameters for CIP removal by the waste-based ceramsite; Table S5: Isotherm model parameters for CIP removal by the waste-based ceramsite.
