# Supplementary material for: Waste-Based Ceramsite for the Efficient Removal of Ciprofloxacin in Aqueous Solutions
Source: Int J Environ Res Public Health. 2023 Mar 13;20(6):5042. doi: 10.3390/ijerph20065042 (PMC10049662; doi:10.3390/ijerph20065042)
Supplement: Supplementary file 1 [file ijerph-20-05042-s001.zip › ijerph-2240995-supplementary.pdf]

Supplementary Materials for

Waste-based ceramsite for the efficient removal of ciprofloxacin in aqueous solutions

Juan Qin[1,*], Yeting Fang[1], Jian Shi[2,*], Chiharu Tokoro[3,4], Mauricio Córdova-Udaeta[3], Keishi Oyama[3] and Juncheng Zhang[5]

[1] Nantong Key Laboratory of Intelligent and New Energy Materials, School of Chemistry and Chemical Engineering, Nantong University, Nantong 226019, China

[2] Analysis and Testing Center, Nantong University, Nantong 226019, China

[3] Faculty of Science and Engineering, Waseda University, 3-4-1 Okubo, Shinjuku-ku, Tokyo 169-8555, Japan

[4] Faculty of Engineering, University of Tokyo, 7-3-1 Hongo, Bunkyo-ku, Tokyo 113-8656, Japan

[5] Department of Science and Engineering, Aoyama Gakuin University, Sagamihara 252-5258, Japan

* Correspondence: qinjuan880816@ntu.edu.cn (J.Q.); Shi.j1@ntu.edu.cn (J.S.)

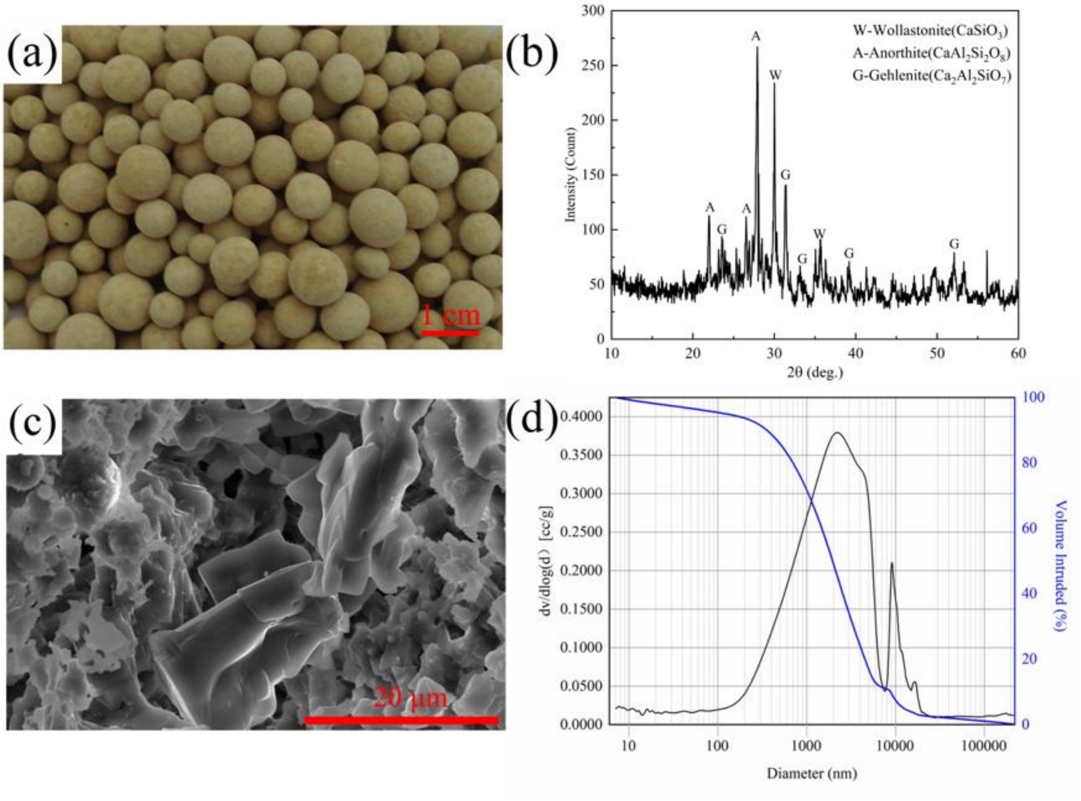

Figure S1. (a) Photograph, (b) XRD pattern, (c) SEM image and (d) pore size distribution of the waste-based ceramsite.

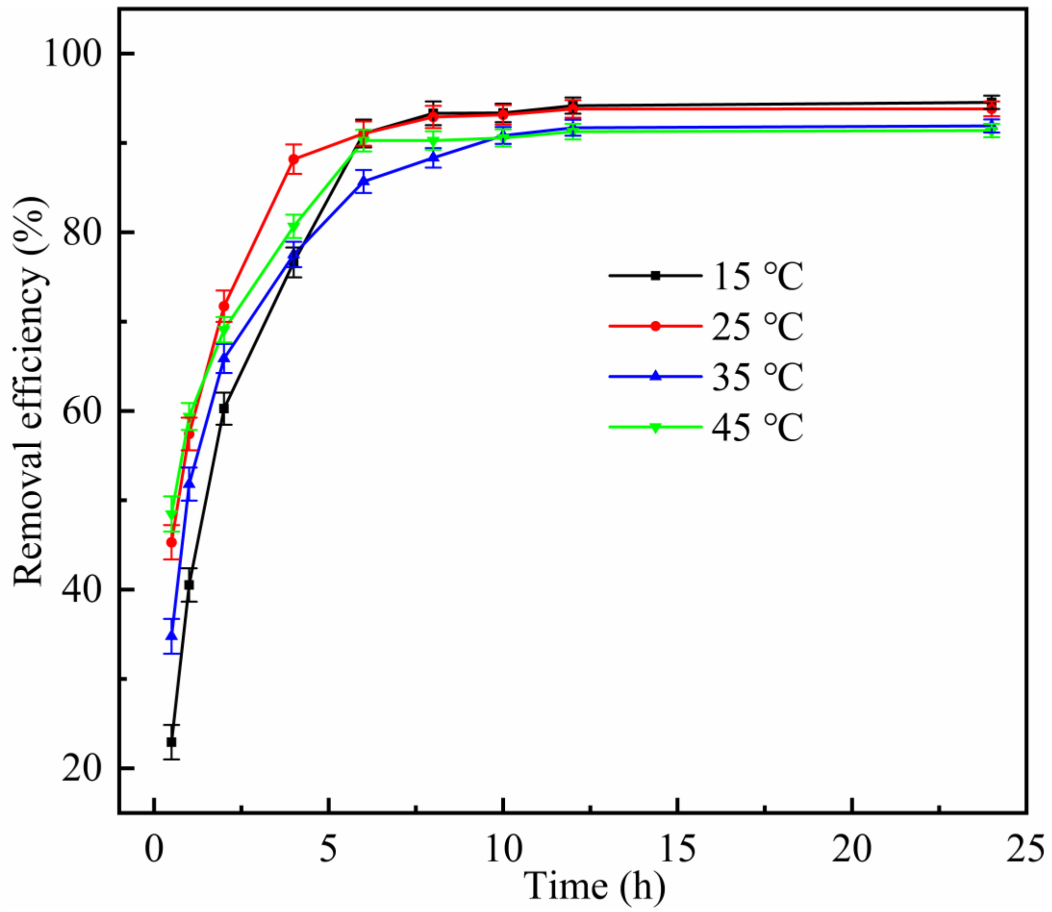

Figure S2. Removal efficiency of CIP at different temperature (initial pH: 2.0; ceramsite dosage: 6 g/200 mL; CIP concentration: 100 mg/L).

Table S1. Concentrations of typical anions and antibiotics in the influent of a local sewage treatment plant.

**Anion (mg/L)**			**Antibiotic (ng/L)**				
**Cl^-**	**NO_3^-**	**SO_4^{2-}**	**Lincomycin**	**Ofloxacin**	**Azithromycin**	**Ciprofloxacin**	**Norfloxacin**
541.27	0.59	37.91	338.54	192.58	131.70	71.46	60.08

Table S2. Physical properties of the waste-based ceramsite.

**Bulk density (g/cm^3)**	**24h-water adsorption (%)**	**Apparent porosity (%)**	**Cylinder compressive strength (MPa)**
0.88	30.15	48.57	2.43

Table S3. Results of leaching test for the waste-based ceramsite.

**Item**	**Leaching concentration (mg/L)**	**Limited values (mg/L)**	
	**Ceramsite**	**United States[1]**	**China[2]**
As^{3+}	0.0140	5	5
Hg^{2+}	ND[3]	0.2	0.1
Ag^{+}	ND	5	5
Se	0.0340	1	1
Cr^{3+}	0.1956	5	15
Cd^{2+}	0.4331	1	1
Cu^{2+}	0.0205	NS[4]	100
Pb^{2+}	0.6742	5	5
Zn^{2+}	0.1024	NS	100
Ni^{2+}	1.3771	NS	5
Be^{2+}	0.0107	NS	0.02
Ba^{2+}	0.1458	100	100

1 Taken from U.S. Government Publishing Office. 40 CFR 261.24 - Toxicity characteristics

2 Taken from GB 5085.3-2007

3 ND = Not detected

4 NS = Not specified

Table S4. Kinetic model parameters for CIP removal by the waste-based ceramsite.

$Q_{e,exp}$	Pseudo-first-order			Pseudo-second-order		
	$Q_{e,cal}$	K_1	R^2	$Q_{e,cal}$	K_2	R^2
1.995	1.926	0.821	0.967	2.117	0.545	0.994

Table S5. Isotherm model parameters for CIP removal by the waste-based ceramsite.

Langmuir			Freundlich		
Q_m	K_L	R^2	n	K_F	R^2
10.079	0.068	0.916	0.355	1.783	0.982
